# Plasma-Activated Saline Promotes Antibiotic Treatment of Systemic Methicillin-Resistant *Staphylococcus aureus* Infection

**DOI:** 10.3390/antibiotics10081018

**Published:** 2021-08-22

**Authors:** Lu Yang, Gulimire Niyazi, Yu Qi, Zhiqian Yao, Lingling Huang, Zifeng Wang, Li Guo, Dingxin Liu

**Affiliations:** 1 School of Life Science and Technology, Xi’an Jiaotong University, Xi’an 710049, China; yanglu35@stu.xjtu.edu.cn (L.Y.); gulmira@stu.xjtu.edu.cn (G.N.); 2 State Key Laboratory of Electrical Insulation and Power Equipment, Center for Plasma Biomedicine, Xi’an Jiaotong University, Xi’an 710049, China; qq460820339@stu.xjtu.edu.cn (Y.Q.); yaozhiqian0512@stu.xjtu.edu.cn (Z.Y.); huangll@stu.xjtu.edu.cn (L.H.); zsdgsd@stu.xjtu.edu.cn (Z.W.)

**Keywords:** cold atmospheric-pressure plasma, plasma-activated saline, methicillin-resistant *Staphylococcus aureus*, systemic infection, biofilm

## Abstract

Systemic infections caused by methicillin-resistant *Staphylococcus aureus* (MRSA) are life-threatening due to their strong multidrug resistance, especially since the biofilms formed by MRSA are more difficult to inactivate by antibiotics, causing long term recurrence of infection. Plasma-activated saline (PAS), a derived form of cold atmospheric-pressure plasma, can effectively inactivate bacteria and cancer cells and has been applied to sterilization and cancer treatment. Previous studies have demonstrated that the pretreatment of MRSA with PAS could promote the action of antibiotics. Here, the PAS was used as an antibiotic adjuvant to promote the inactivation of MRSA biofilms by rifampicin and vancomycin, and the combined treatment reduced approximately 6.0-log_10_ MRSA cells in biofilms. The plasma-activated saline and rifampicin synergistically and effectively reduced the systemic infection in the murine model. The histochemical analysis and the blood hematological and biochemical test demonstrated that the combined treatment with plasma-activated saline and rifampicin improved the blood hematological and biochemical parameters of infected mice by reducing the infection. Therefore, PAS based on plasma technology represents a new strategy for the treatment of infectious disease caused by multidrug-resistant bacteria and alleviating antibiotic resistance.

## 1. Introduction

Methicillin-resistant *Staphylococcus aureus* (MRSA) is a considerably antibiotic-resistant bacteria of hospital-acquired and community-acquired infections [1,2,3]. One type of serious infection caused by MRSA is systemic infections, which lead to organ failure and death [4,5]. MRSA generally exists in the form of a biofilm in the lesion location, and it is not easy for the living immune system to inactivate it [6,7,8,9,10,11]. Due to the strong antibiotic resistance, MRSA biofilm is difficult to inactivate with a low concentration of antibiotics, and a higher concentration of antibiotics would cause renal toxicity and are almost impossible to achieve in vivo [12,13,14,15]. The development and approval of new antibiotics are arduous and antibiotic resistance may emerge soon after the clinical application of new antibiotics, which is shorter than the time required for antibiotic research [16,17,18]. Therefore, the development of new strategies is necessary to improve the inactivating effects of available antibiotics on antibiotic-resistant bacteria for the effective treatment of infectious diseases [12,16,19].

A common clinical strategy is the combination of different types of antibiotics. Rifampicin combination is recommended for the treatment of prosthetic valve infective endocarditis and prosthetic joint infections by the Infectious Diseases Society of America. A previous study demonstrated that the combination of rifampin with one of the other four different antibiotics, vancomycin, linezolid, daptomycin, and ceftaroline, respectively, was used to treat periprosthetic joint infection in a murine model caused by MRSA and entirely reduced the MRSA in the infection focus of mice [20]. However, the interactions between antibiotics may cause side effects in the combination of antibiotics and the accumulation of antibiotics also brings a high risk of cytotoxicity [16,21]. Another strategy is the combination of adjuvants with antibiotics, which can enhance the bactericidal effect of antibiotics [22,23]. Lysophosphatidylcholine, as an antibiotic adjuvant, was combined with colistin, and the combination inactivated more than 6-log_10_ bacteria in murine peritoneal sepsis and pneumonia models caused by *Acinetobacter baumannii* and improved the survival rate by more than 75% [24]. Seven antibiotics were combined with Ag-nanoparticles to treat *Escherichia coli* and MRSA infections in rats and significantly reduced the infection after three-week combinational treatment [25]. The combined application of antibiotics and adjuvants is still in its infancy and the development of new adjuvants is an important strategy for alleviating antibiotic resistance.

Cold atmospheric-pressure plasma (plasma for short) produces a variety of reactive species, such as H_2_O_2_ and O_2_^•‒^, which could efficiently inactivate a variety of microorganisms [26,27,28,29]. The most common structures of cold atmospheric-pressure plasma generation are the surface dielectric barrier discharge (SDBD) and the atmospheric pressure plasma jet (APPJ) [30,31,32]. SDBD can generate large-scale and stable plasmas on the surface of the dielectric, and the reactive species in plasma diffuse into the downward treated samples [33,34]. The plasma was generated by APPJ by ionizing the gas near the electrode and the reactive species was blown out with the gas flow and formed a plasma plume with a length up to several centimeters [32,35]. Plasma-activated water, as a derived form of cold atmospheric-pressure plasma, is prepared using water treated with both SDBD and APPJ plasma devices. Plasma-activated water also exhibits excellently bactericidal ability and can be injected and sprayed, which greatly expands the applications of plasma [36,37,38,39]. The plasma-activated water prepared by the helium or argon plasma jet for 50 min inactivated approximately 40–45% of *S. aureus* after incubation with bacteria suspensions for 24 h and that prepared with air plasma jet for 10 min inactivated approximately 6-log_10_ *S. aureus* in the presence of bovine serum albumin (BSA) and more than 7-log_10_ in the absence of BSA [40,41]. For the treatment of biofilm, Charoux et al. found that plasma-activated water prepared by plasma jet could inactivate 1.79-log_10_ cells of *E. coli* biofilm [42]. When plasma-activated water, which is prepared by activating deionized water with plasma, is applied to cells or living bodies it affects the osmotic pressure. Therefore, other solutions, such as normal saline or medium, instead of water, can be used to prepare plasma-activated normal saline (PAS) or plasma-activated medium for application on cells or living bodies [43,44]. Takeda et al. demonstrated that the treatment of plasma-activated liquid could reduce the migration and adhesion of gastric cancer cells in vitro, and intraperitoneal plasma-activated liquid could reduce the formation of peritoneal metastatic nodules by 60% in a murine model [45]. A previous study demonstrated that the pretreatment of MRSA cells with PAS activated by plasma for a short time could promote antibiotic sensitivities and enhance the inactivation of antibiotics [46]. However, the application of the combination of PAS and antibiotics on infectious diseases, especially systemic infections, has not been reported.

In this study, PAS activated by surface discharge plasma was used to combine with antibiotics for the treatment of MRSA biofilms. The inactivation effects of the combination of the PAS and antibiotics were investigated both in vitro and in the murine model. The combined treatment of PAS and antibiotics provides a novel strategy for the treatment of MRSA infections.

## 2. Results

### 2.1. Reactive Species in PAS

**Figure 1:**
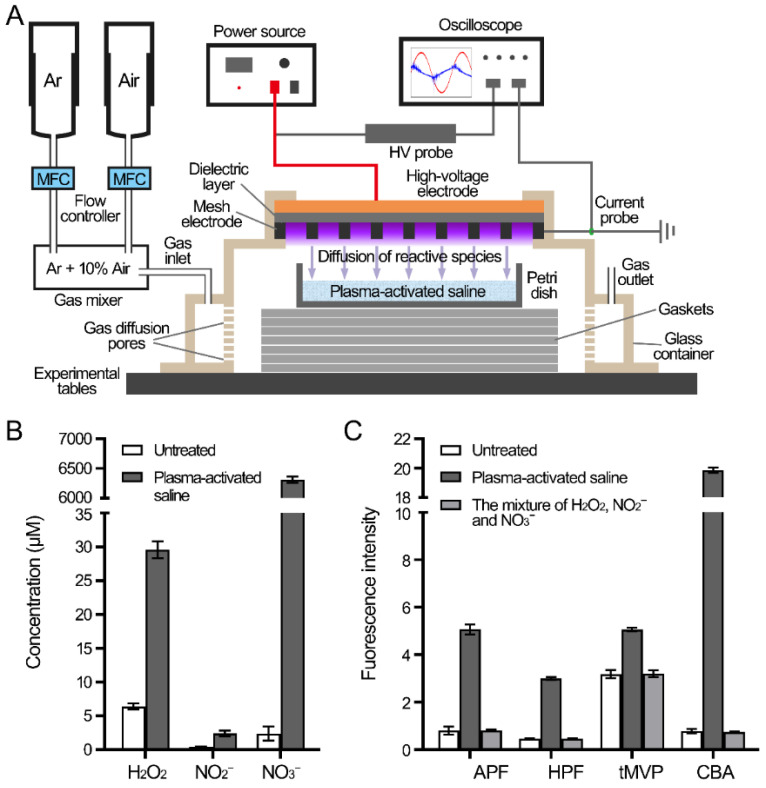
Reactive species in the PAS. (**A**) Diagram of the device for the preparation of PAS. (**B**) The concentrations of three long-lived species H_2_O_2_, NO_2_^−^, and NO_3_^−^ in PAS. (**C**) The fluorescence intensities of the probes in PAS. Four probes APF, HPF, tMVP, and CBA were mixed with the PAS and untreated saline, then the fluorescence intensities were measured.

The gas near the mesh electrode of the surface plasma was broken down by a high voltage to generate plasma, and the reactive species of plasma diffused into the underlying normal saline to produce PAS (Figure 1A). First, the reactive species in PAS were determined through measurements of the concentrations of three long-lived species and the fluorescence intensities of four probes for short-lived species (Figure 1B,C). The concentration of H_2_O_2_ in the PAS was 29.6 μM, while the concentrations of NO_2_^−^ and NO_3_^−^ were 2.4 μM and 6312.0 μM, respectively. Four fluorescence probes APF for ^•^OH, ONOO^−^, and ClO^−^, HPF for ^•^OH and ONOO^−^, tMVP for ^1^O_2_, and CBA for ONOO^−^ were used to detect the relative levels of short-lived species. Compared with the probes in the untreated saline, the fluorescent intensities of APF and HPF in PAS increased by 4.3 and 2.5, respectively, while those of tMVP and CBA increased by 1.9 and 19.1, respectively. The fluorescent intensities of the probes in the artificial mixture of three long-lived H_2_O_2_ (30 μM), NO_2_^−^ (2.5 μM), and NO_3_^−^ (6500 μM) exhibited similar levels to those in the untreated saline, therefore, the existence of short-lived species in the PAS contributed to the increase in fluorescent intensities. The results demonstrated that a variety of long-lived and short-lived reactive species existed in the PAS.

### 2.2. Biofilm Reduced by the Combination of PAS and Antibiotics In Vitro

**Figure 2:**
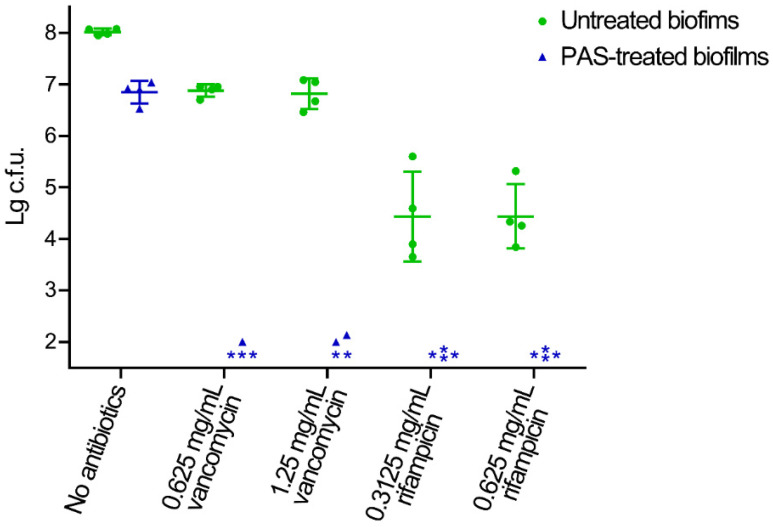
Inactivation of MRSA biofilms by the combination of PAS and antibiotics in vitro. The PAS-treated and untreated biofilms were incubated with TSB broth containing vancomycin or rifampicin. The treated and untreated biofilms were solubilized in 1 mL PBS by sonication and vortexing. Serial dilutions of each biofilm were performed and 10 μL of each dilution was spotted onto TSB plates and incubated overnight at 37 °C. The resulting colonies were counted and analyzed. The asterisks represent when the numbers of the colonies were less than the detection limit. Error bars represent the standard deviation (SD).

The inactivation effects of the combination of PAS and vancomycin or rifampicin on the MRSA biofilms in vitro were investigated (Figure 2). There were approximately eight orders of magnitude of MRSA cells in the biofilm cultured on 1 cm^2^ silica gel after three days of culturing. The treatment of PAS alone inactivated approximately 1.2 orders of magnitude of MRSA cells, and the treatment of vancomycin or rifampicin alone inactivated approximately 1.2 and 3.6 orders of magnitude of MRSA cells in the biofilm, respectively. The minimum bactericidal concentrations (MBC) of vancomycin and rifampicin were >2.5 mg/mL and 2.5 mg/mL, respectively, therefore, more than two times lower concentrations of vancomycin (0.625 mg/mL or 1.25 mg/mL) and rifampicin (0.3125 mg/mL or 0.625 mg/mL) were used to analyze the effects of the PAS. The combined treatment of PAS and vancomycin or rifampicin reduced approximately six orders of magnitude and more than six orders of magnitude MRSA cells in the biofilms, respectively. The reconstituted saline solution with three long-lived species (30 μM H_2_O_2_, 2.5 μM NO_2_^−^ and 6500 μM NO_3_^−^) in saline solution were used as control and exhibited little effect in the inactivation of biofilms and the promotion of antibiotics in vitro (Figure S1).

### 2.3. Treatment with PAS and Rifampicin in a Murine Systemic Infection Model

**Figure 3:**
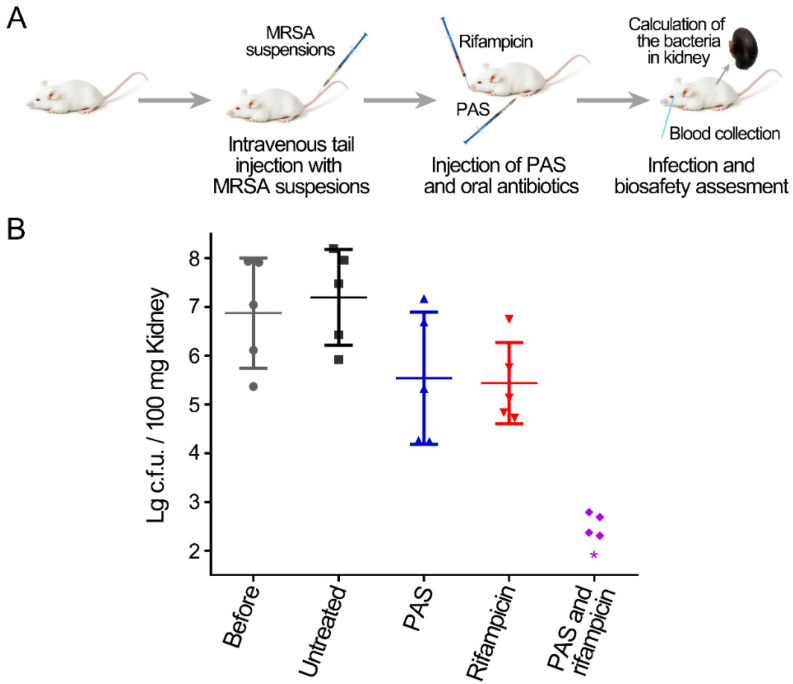
Treatment of MRSA systemic infection by the combination of PAS and rifampicin in the murine model. (**A**) Schematic representation of the wound model and PAS and antibiotic treatment. (**B**) MRSA systemic infection improved by the combined treatment of PAS and rifampicin. Groups of five Balb/c mice were used. Colony-forming units (c.f.u.) from one kidney of each mouse were plotted as individual points and error bars represent the standard deviation (SD) within an experimental group. Gray circles, the infected group before treatment; black squares, the untreated group; blue regular triangles, the group treated with the PAS; red inverted triangles, the group treated with rifampicin; purple rhombuses, the group treated with the combination of the PAS and rifampicin. The asterisks represent that the number of the colonies was less than the detection limit.

Based on the inactivation effect of in vitro assay, the combination treatment of PAS and antibiotics was applied to the murine MRSA systemic infection model (Figure 3A). Three days after an intravenous tail injection with MRSA cells, the infection levels of the lungs, livers, spleens, and kidneys of mice were compared. There were approximately four orders of magnitude of MRSA cells in the lungs, livers, and spleens, and approximately seven orders of magnitude of MRSA cells in the kidneys (Figure S2). Therefore, the bacterial accumulation in systemic infection was mainly in the kidneys, and the numbers of MRSA in the kidneys were mainly detected to evaluate the effects of different treatments. The treatment of PAS or rifampicin alone reduced less than two orders of magnitude of MRSA cells, while the combined treatment of PAS and rifampicin reduced approximately five orders of magnitude of MRSA cells in the kidneys of systemic infectious mice (Figure 3B). Similarly, the reconstituted saline solution exhibited no effect on the systemic infection in the murine model (Figure S3). The results suggested that the combination of PAS and antibiotics could effectively reduce the MRSA cells in the systemic infection model.

### 2.4. Biosafety of the Combination Treatment in a Mice Model

**Figure 4:**
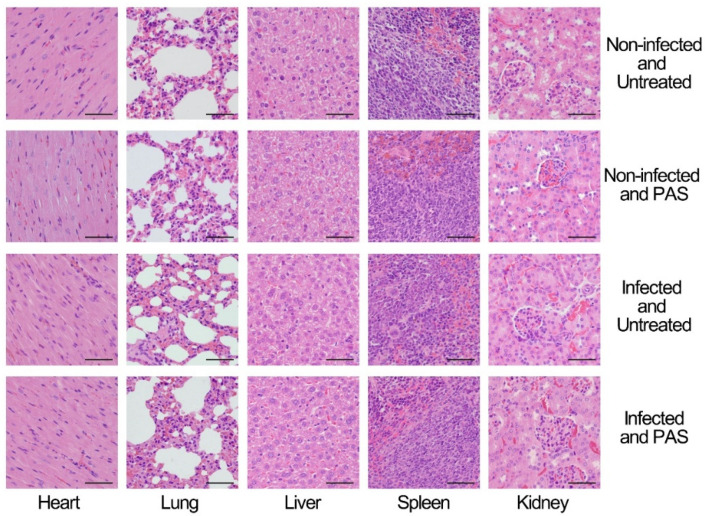
Histopathological analysis of the hearts, lungs, livers, spleens, and kidneys of the mice. The tissues were fixed with paraformaldehyde solution and compared after hematoxylin and eosin (H&E) staining. The bar represents 50 μm.

As a treatment strategy, the biosafety of PAS is the primary consideration. In the assessment of biosafety, to get rid of the impact of infection, two groups, non-infected mice and non-infected mice treated with PAS or the combined treatment of PAS and rifampicin, were assigned as the control. In the hematological test, the treatment of PAS or the combined treatment of PAS and rifampicin caused little effect on the non-infected mice, while the infection of MRSA caused an increase in the numbers of white blood cells and platelets and the percentage of neutrophils and monocytes and a decrease in the percentage of lymphocytes. After the treatment of PAS or the combined treatment of PAS and rifampicin on the infected mice, the numbers of white blood cells and the percentage of neutrophils, and monocytes decreased and the percentage of lymphocytes increased to the levels of the non-infected mice, but the numbers of platelets exhibited little change (Table 1). In the biochemical test of the serum, the treatment of PAS or the combined treatment of PAS and rifampicin on the non-infected mice also had little effect. The infection of MRSA caused an increase in uric acid and decreases in alkaline phosphatase, albumin, glycosylated serum protein, and triglyceride. After the treatment of PAS or the combined treatment of PAS and rifampicin on the infected mice, the level of uric acid decreased and that of glycosylated serum protein increased, but still had a difference with those in non-infected mice, while the levels of alkaline phosphatase, albumin, and triglyceride exhibited few changes (Table 2). Furthermore, the histochemical analysis of the hearts, lungs, livers, spleens, and kidneys, which are commonly used for toxicological tests, from mice was conducted. The histological analysis demonstrated that there was no significant change in cellular swelling, fatty degeneration, hyaline change, or other pathological damage in the organs after the treatment of PAS in both non-infected and infected groups (Figure 4). These results indicated that the treatment of PAS exhibited little effect on the blood parameters in the non-infected mice and improved the blood parameters in the infected mice.

## 3. Discussion

Systemic infections caused by methicillin-resistant *S. aureus* exhibit high mortality, and only a few antibiotics, such as linezolid and daptomycin, are available due to its increasing resistance to vancomycin [47,48,49,50,51,52]. The development of new therapeutic strategies is urgent for infectious diseases caused by multidrug-resistant bacteria [53,54,55]. In this study, the combination of PAS, a derived form of cold atmospheric-pressure plasma, and antibiotics effectively reduced the MRSA biofilms in vitro and lower the number of MRSA cells in a murine systemic infection model.

How did the PAS synergistically act with antibiotics? This effect may be realized in two ways. The first way was that the reactive species in PAS directly entered into the MRSA cells and inactivated MRSA. In the in vitro assay, the reactive species in PAS diffused into the biofilms and directly acted on the MRSA cells with high concentrations at the beginning. The reactive species entered the MRSA cells and inevitably caused oxidative damage of biomacromolecules, such as protein and DNA, resulting in the death of MRSA cells [56,57,58,59]. This partially contributed to the inactivation effect of the combined treatment, which was proven by the fact that the treatment of PAS alone reduced approximately 1.2 orders of magnitude of MRSA (Figure 3). The second way was that the pretreatment of PAS with less reactive species promoted the inactivation of MRSA by antibiotics. The previous study by our group demonstrated that the pretreatment of MRSA with PAS activated with plasma for 30 s could promote the sensitivities to several antibiotics and enhanced inactivation by antibiotics [44,46]. When the reactive species in PAS were reduced and acted on MRSA cells, PAS was not sufficient to inactivate MRSA but could sensitize the MRSA to promote their killing by antibiotics. Based on these two mechanisms, PAS and antibiotics could synergistically effectively reduce the MRSA cells in biofilms (Figure 3).

In the murine model, the effect of PAS was complicated due to the complex biological systems. At present, studies on the application of plasma-activated liquid on infectious disease are focused on the treatment of wound infections, however, the injection of plasma-activated liquid for the treatment of internal infection has not been reported [60,61]. Several previous studies have demonstrated that plasma-activated medium produced by the dielectric barrier discharge device with the working gas of oxygen and air could induce the death of immunogenic cells by activating the innate immune system [32,62,63]. These studies demonstrated that the reactive species of PAS could work in the biological system. It was speculated that the effects of PAS were not strong enough to kill the bacteria in vivo. Thus, the treatment with PAS alone for the systemic infection reduced approximately 1.6 orders of magnitude of MRSA cells in the kidneys. However, the effect of PAS in vivo on the MRSA cells could promote the inactivation effects of antibiotics, and the combined treatment reduced the MRSA cells close or lower than the detection limit. Therefore, the application of PAS in vivo could promote the action of antibiotics for the treatment of infectious diseases caused by antibiotic-resistant bacteria. In this study, both the in vitro and in vivo analyses of the combined treatment of PAS and antibiotics were only for MRSA biofilms. However, the effects of the combined treatment for other multidrug-resistant bacteria or fungi are still unknown, and more studies are needed to verify this.

Due to the non-selectivity of the reactive species in PAS, as the PAS was used as an antibiotic adjuvant, biosafety must be taken into account. The non-infected mice treated with PAS exhibited few effects on the hematological and serum biochemical parameters. The MRSA-infected mice treated with PAS showed reduced numbers of MRSA cells; restored numbers of white blood cells and percentages of neutrophils, lymphocytes, and monocytes; and two serum biochemical parameters were restored to the normal levels (Table 1 and Table 2). The histological analysis also exhibited that the PAS treatment had little effect on the organs (Figure 4). These analyses shed light on the biosafety of PAS treatment on the murine model. The PAS is prepared by the treatment of saline with plasma, which is produced by the ionization of the gas. Therefore, the reactive species in PAS are all small and inorganic, such as hydrogen peroxide and peroxynitrite, most of which also exist in living organisms [64,65,66]. Therefore, the application of PAS does not produce secondary residues or cause residue accumulation or renal toxicity.

**Figure 5:**
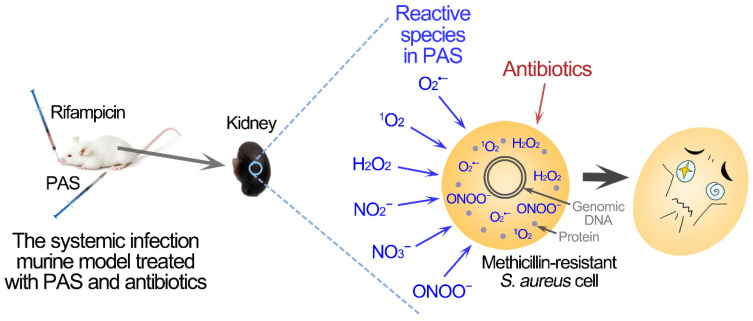
The systemic methicillin-resistant *S. aureus* infection treated with the combined treatment of PAS and antibiotics.

Based on these results, a strategy of treatment for systemic infection caused by methicillin-resistant *Staphylococcus aureus* was proposed (Figure 5). Plasma-activated saline was prepared by cold atmospheric-pressure plasma and contained multiple reactive species, such as singlet oxygen and peroxynitrite. The combined treatment of PAS and vancomycin or rifampicin synergistically reduced the MRSA cells in biofilms. The combination therapy was also effective and safe in the treatment of systemic infection in the murine model. The PAS treatment of the physical method supplied an interdisciplinary strategy to alleviate antibiotic resistance and the combination of PAS and antibiotics could be developed into a potential therapy for systemic infections.

## 4. Materials and Methods

### 4.1. Plasma Device and Preparation of Plasma-Activated Saline

As shown in Figure 1A, the surface discharge structure of the plasma consisted of a high-voltage plane electrode, a liquid-facing grounded mesh electrode of hexagonal shape, and a dielectric layer (made of alumina ceramics) sandwiched between the two electrodes. Plasma (80 mm × 80 mm) was generated when a sinusoidal high voltage was applied, and the discharge power density was 0.2 W/cm^2^ with good mesh-to-mesh homogeneity (showed in the front view of the plasma in Figure 1A). Five milliliters of saline in a Petri dish (70 mm × 70 mm made of quartz glass), which was smaller than the area of the surface plasma with a depth of 1 mm, was placed under the plasma. The air gap between the plasma and the liquid surface was about 8 mm. The gas plasma system was housed in a sealed organic glass box with a gas flow of synthetic air (79% N_2_ + 21% O_2_) at a constant flow rate of 4 L/min. The normal saline was treated with surface discharge plasma for 10 min to produce PAS, then PAS was transferred into 15-mL Corning tubes and kept at room temperature.

### 4.2. Measurement of Reactive Species

The concentrations of long-lived H_2_O_2_ and NO_2_^−^/NO_3_^−^ in PAS were determined by using a hydrogen peroxide/peroxidase assay kit and nitrite/nitrate colorimetric assay kit (Beyotime Biotechnology, Shanghai, China), respectively. The short-lived species were detected by using fluorescent probes, 3′-(p-aminophenyl) fluorescein (APF, Sigma), 3′-(p-hydroxyphenyl) fluorescein (HPF, Sigma), trans-1-(2′-methoxyvinyl) pyrene (tMVP, J&K Scientific, Beijing, China), and coumarin boronic acid pinacolate ester (CBA, Cayman, Michigan, USA) at final concentrations of 5 μM, 5 μM, 10 μM, and 20 μM, respectively. The fluorescent probes were mixed with PAS thoroughly and incubated for 10 min at room temperature. After that, the fluorescence intensities were measured using a microplate reader (Thermo Scientific Varioskan Flash, Waltham, Massachusetts, USA) at the corresponding excitation and emission wavelengths (APF and HPF: 490/515 nm; tMVP: 405/460 nm; CBA: 332/470 nm).

### 4.3. Biofilm Assay

*Staphylococcus aureus* ATCC33591, a methicillin-resistant strain, was purchased from the American Type Culture Collection (ATCC, Maryland, USA). The culture of biofilms was performed as described previously [67]. After culturing for three days, the medium was carefully removed, and the silica films with biofilms were gently washed three times with saline solution (0.9% NaCl). After that, the silica films with biofilms were incubated with PAS for 30 min and then were cultured in the presence of 0.625 mg/mL and 0.3125 mg/mL rifampicin or 1.25 mg/mL and 0.625 mg/mL vancomycin in TSB with 1% glucose at 37 °C for 24 h. Then, the biofilms were solubilized in 1 mL phosphate-buffered saline (PBS) in 1.5 mL Eppendorf tubes by sonication for 1 min and vortex for 5 min. Serial dilutions of each biofilm were performed and 10 μL of each dilution was spotted onto TSB plates and incubated overnight at 37 °C. The numbers of surviving MRSA were calculated by colony-forming unit assay (c.f.u.). The detection limit in this study was 10^2^ cells/mL.

### 4.4. Mouse Systemic Infection Model

All the animal experiments were approved and supervised by the laboratory animal care committee of Xi’an Jiaotong University, Xi’an, China. A single *S. aureus* ATCC33591 colony was grown in 4 mL of Tryptic Soy Broth (TSB) broth (Oxoid) at 225 r.p.m. at 37 °C overnight, and the resulting overnight culture was diluted 1:100 in TSB and incubated at 37 °C at 225 r.p.m. until an OD_600_ of 0.6 was reached. The bacterial cells were collected by centrifugation, washed once with phosphate-buffered saline (PBS), and resuspended in PBS at an OD_600_ of 0.5. Mice were inoculated by injection of a 100 μL MRSA suspension into the lateral tail vein (final amount, 2 × 10^7^ c.f.u. per mouse) through a 26-gauge needle. The preliminary experiments demonstrated that the challenge dose of *S. aureus* effectively caused systemic infection and did not induce the death of the mice in the experiment cycle (data not shown). The actual bacterial numbers of *S. aureus* were also verified by serial 10-fold dilutions and spotted on TSB agar. The day of the challenge was designated as day 1 of the experiment.

### 4.5. Treatment

From day 2 to day 4, 150 μL of PAS was administered by intraperitoneal injection and rifampicin (30 mg/kg) was given by intragastric administration once a day. On day 5, the blood of mice was collected from the eyes using a capillary. The blood was divided into two samples: 0.2 mL was stored in anticoagulant tubes for the hematological test, and the rest was stored at 4 °C overnight then the serum was separated by centrifugation for the detection of serum biochemical markers. After the collection of blood, the mice were sacrificed by dislocation of the cervical vertebra, and the lung, heart, liver, spleen, and one kidney of each mouse were removed and stored in 4% paraformaldehyde solution to be used for hematoxylin and eosin (H&E) staining. The other kidneys of the mice were added to 900 μL PBS in 2 mL Eppendorf tubes and homogenized in PBS using a homogenizer (IKA). Homogenates were serially diluted and samples were plated on TSB agar and incubated at 37 °C overnight. The bacteria numbers were determined by counting the resulting CFUs. The blood was detected using an automatic animal blood analyzer (Minrdray BC-2800Vet, Shenzhen, China) and the serum samples were diluted two times and measured using an automatic biochemical analyzer (Chemray 800, Shenzhen, China).

### 4.6. Statistical Analysis

All the experiments were performed independently at least three times. Statistical analyses were performed in SPSS 13.0 (IBM, Armonk, NY, USA) using the Mann–Whitney *U* test, a non-parametric statistical test. The statistical significance of the data was established at a *p*-value of <0.05.

## 5. Conclusions

In the study, the biofilms formed by methicillin-resistant *S. aureus* (MRSA) were effectively reduced by the combination of PAS and rifampicin or vancomycin. Further, the combination of PAS and rifampicin could reduce the MRSA infection in the systemic infection murine model. The biosafety analysis demonstrated the treatment of PAS alone or the combination of PAS and rifampicin improved the blood hematological and biochemical parameters of infected mice by reducing the infection. Therefore, plasma-activated saline based on the cold atmospheric-pressure plasma technology provided a new strategy for the treatment of infectious disease caused by multidrug-resistant bacteria and alleviating antibiotic resistance.

## Figures and Tables

**Table 1:** Effects of PAS on hematological parameters in mice.

Parameter	Non-Infected	Non-Infected and Treated with PAS	Non-Infected and Treated with PAS and Rifampicin	Infected	Infected and Treated with PAS	Infected and Treated with PAS and Rifampicin
RBC (×10^12^/L)	11.23 ± 0.56	11.33 ± 0.88	11.44 ± 0.31	11.23 ± 0.94	11.68 ± 0.64	11.14 ± 0.72
WBC (×10^9^/L)	5.32 ± 0.87	7.28 ± 2.70	6.48 ± 1.25	7.94 ± 0.87 *^a^*	5.42 ± 1.37 *^b^*	5.56 ± 1.13 *^b^*
Neutrophils (%)	36.62 ± 6.47	32.32 ± 6.62	36.38 ± 4.86	50.70 ± 8.02 *^a^*	38.56 ± 3.46 *^b^*	31.56 ± 6.53 *^b^*
Lymphocytes (%)	59.62 ± 7.12	63.72 ± 6.74	60.76 ± 4.83	42.74 ± 8.53 *^a^*	57.00 ± 3.64 *^b^*	65.06 ± 6.35 *^b,c^*
Monocytes (%)	3.76 ± 0.88	3.96 ± 0.89	3.88 ± 0.96	6.56 ± 1.16 *^a^*	4.44 ± 0.29 *^b^*	4.58 ± 0.68 *^b^*
MCV (fL)	49.80 ± 0.95	50.22 ± 1.04	49.42 ± 0.51	49.08 ± 0.70	48.84 ± 0.94	48.46 ± 1.35
MCH (pg)	14.90 ± 0.39	14.84 ± 0.28	14.76 ± 0.19	14.62 ± 0.45	14.68 ± 0.36	14.64 ± 0.32
MCHC (g/L)	299.80 ± 3.11	296.60 ± 7.13	299.00 ± 0.71	296.00 ± 2.83	302.00 ± 7.52	304.40 ± 7.54
PLT (×10^9^/L)	1330.0 ± 177.5	1455.2 ± 201.4	1584.6 ± 87.9	2480.0 ± 277.8 *^a^*	2613.0 ± 258.6 *^a^*	2649.4 ± 620.3 *^a^*
HGB (g/L)	168.0 ± 11.9	168.8 ± 14.7	169.2 ± 4.3	162.6 ± 14.0	172.4 ± 11.2	163.8 ± 11.3
Hematocrit (%)	55.92 ± 3.74	56.80 ± 4.15	56.46 ± 1.52	54.62 ± 4.43	57.02 ± 3.79	54.00 ± 4.51

HGB, hemoglobin; MCH, mean corpuscular hemoglobin; MCHC, mean corpuscular hemoglobin concentration; MCV, mean corpuscular volume; PLT, platelet count; RBC, red blood cells; WBC, white blood cells; *^a^* *p* < 0.05, comparison with the non-infected group (determined by the Mann–Whitney *U* test). *^b^ p* < 0.05, comparison with the infected group (determined by the Mann–Whitney *U* test). *^c^ p* < 0.05, comparison with the infected and treated with PAS group (determined by the Mann–Whitney *U* test).

**Table 2:** Effects of PAS treatment on serum biochemical parameters in mice.

Parameter	Non-Infected	Non-Infected and Treated with PAS	Non-Infected and Treated with PAS and Rifampicin	Infected	Infected and Treated with PAS	Infected and Treated with PAS and Rifampicin
Liver						
AST (U/L)	144.24 ± 10.78	163.72 ± 23.42	151.73 ± 11.97	169.40 ± 31.74	161.17 ± 15.46	163.09 ± 12.91
ALT (U/L)	49.76 ± 13.00	48.17 ± 6.43	56.36 ± 14.01	50.37 ± 8.31	58.68 ± 8.27	58.82 ± 3.75
ALP (U/L)	189.18 ± 27.75	161.47 ± 22.69	176.53 ± 34.60	93.60 ± 29.38 *^a^*	123.20 ± 13.36 *^a^*	125.35 ± 27.03 *^a^*
T-BIL (μmol/L)	21.32 ± 5.99	22.34 ± 3.89	21.70 ± 1.99	15.96 ± 3.13	16.96 ± 3.44	21.96 ± 7.25
ALB (g/L)	39.24 ± 2.20	37.26 ± 2.03	37.51 ± 2.55	32.53 ± 1.94 *^a^*	34.40 ± 1.21 *^a^*	36.66 ± 1.39 *^b^*
Kidney						
BUN (mmol/L)	25.79 ± 2.90	24.71 ± 3.30	25.54 ± 3.96	30.41 ± 7.95	24.72 ± 4.11	31.91 ± 6.90
UA (μmol/L)	153.72 ± 38.87	228.70 ± 94.52	202.71 ± 50.36	375.59 ± 48.69 *^a^*	221.07 ± 37.45 *^a,b^*	226.78 ± 97.29 *^b^*
CREA (μmol/L)	24.11 ± 7.70	27.33 ± 5.78	21.45 ± 2.53	31.08 ± 4.11	26.79 ± 1.91	31.96 ± 8.92
Heart						
LDH (U/L)	2300.82 ± 356.61	2835.74 ± 734.15	2795.73 ± 755.79	3125.76 ± 837.71	2698.76 ± 429.21	2918.54 ± 866.03
CK (U/L)	1020.00 ± 276.44	1428.94 ± 458.92	1295.14 ± 170.85	1517.87 ± 514.50	1231.48 ± 224.05	1274.19 ± 154.97
Glucose						
GLU (mmol/L)	1.05 ± 0.50	0.92 ± 0.34	1.21 ± 0.14	0.92 ± 0.63	0.94 ± 0.84	0.97 ± 0.60
GSP (mmol/L)	4.51 ± 0.37	4.50 ± 0.30	4.30 ± 0.50	2.39 ± 0.31 *^a^*	3.32 ± 0.24 *^a,b^*	3.82 ± 0.30 *^a,b,c^*
Lipid						
TG (mmol/L)	1.36 ± 0.51	1.19 ± 0.28	1.25 ± 0.26	0.60 ± 0.13 *^a^*	0.66 ± 0.08 *^a^*	0.66 ± 0.22 *^a^*
T-CHO (mmol/L)	1.74 ± 0.47	2.01 ± 0.19	1.90 ± 0.30	2.41 ± 0.49	1.97 ± 0.29	2.23 ± 0.28
Other						
TP (g/L)	50.06 ± 2.22	53.30 ± 4.48	50.64 ± 1.78	51.32 ± 0.85	50.32 ± 1.67	52.15 ± 5.78

ALB, albumin; ALP, alkaline phosphatase; ALT, alanine aminotransferase; AST, aspartate aminotransferase; BUN, blood urea nitrogen; CK, creatine kinase; CREA, creatinine; GLU, glucose; GSP, glycosylated serum protein; LDH, lactate dehydrogenase; T-BIL, total bilirubin; T-CHO, total cholesterol; TG, triglyceride; TP, total protein; UA, uric acid. *^a^* *p* < 0.05, comparison with the non-infected group (determined by the Mann–Whitney *U* test). *^b^ p* < 0.05, comparison with the infected group (determined by the Mann–Whitney *U* test). *^c^ p* < 0.05, comparison with the infected and treated with PAS group (determined by the Mann–Whitney *U* test).

## Data Availability

Not applicable.

## Supplementary Materials

The following are available online at https://www.mdpi.com/article/10.3390/antibiotics10081018/s1, Figure S1: Inactivation of MRSA biofilms by the combination of the reconstituted saline solution (30 μM H_2_O_2_, 2.5 μM NO_2_^−^, and 6500 μM NO_3_^−^ in saline solution) and antibiotics in vitro. The reconstituted saline solution-treated and untreated biofilms were incubated with TSB broth containing vancomycin or rifampicin. The treated and untreated biofilms were solubilized in 1 mL PBS by sonication and vortexing. Serial dilutions of each biofilm were performed and 10 μL of each dilution was spotted onto TSB plates and incubated overnight at 37 °C. The resulting colonies were counted and analyzed. Error bars represent the standard deviation (SD); Figure S2. Evaluation of the numbers of MRSA cells in different organs from the murine systemic model. Groups of 5 Balb/c mice were used. Colony-forming units (c.f.u.) from the heart, liver, spleen, lung, and kidney of each mouse were plotted as individual points and error bars represent the standard deviation (SD) within an experimental group; Figure S3. Treatment of MRSA systemic infection by the combination of the reconstituted saline solution (30 μM H_2_O_2_, 2.5 μM NO_2_^−^, and 6500 μM NO_3_^−^ in saline solution) and rifampicin in the murine model. Groups of 5 Balb/c mice were used. Colony-forming units (c.f.u.) from one kidney of each mouse were plotted as individual points and error bars represent the standard deviation (SD) within an experimental group.

## References

[1] Chen X., Lou W.Y., Liu J.X., Ding B.S., Fan W.M., Hong J. (2019). A novel antimicrobial polymer efficiently treats multidrug-resistant MRSA-induced bloodstream infection. Biosci. Rep..

[2] Mera R.M., Suaya J.A., Amrine-Madsen H., Hogea C.S., Miller L.A., Lu E.P., Sahm D.F., O’Hara P., Acosta C.J. (2011). Increasing Role of *Staphylococcus aureus* and Community-Acquired Methicillin-Resistant *Staphylococcus aureus* Infections in the United States: A 10-Year Trend of Replacement and Expansion. Microb. Drug Resist..

[3] Panlilio A.L., Culver D.H., Gaynes R.P., Banerjee S., Henderson T.S., Tolson J.S., Martone W.J. (1992). Methicillin-resistant *Staphylococcus aureus* in U.S. hospitals, 1975–1991. Infect. Control Hosp. Epidemiol..

[4] Kokai-Kun J.F., Chanturiya T., Mond J.J. (2007). Lysostaphin as a treatment for systemic *Staphylococcus aureus* infection in a mouse model. J. Antimicrob. Chemoth..

[5] Turner N.A., Sharma-Kuinkel B.K., Maskarinec S.A., Eichenberger E.M., Shah P.P., Carugati M., Holland T.L., Fowler V.G. (2019). Methicillin-resistant *Staphylococcus aureus*: An overview of basic and clinical research. Nat. Rev. Microbiol..

[6] Kumar A., Alam A., Rani M., Ehtesham N.Z., Hasnain S.E. (2017). Biofilms: Survival and defense strategy for pathogens. Int. J. Med. Microbiol..

[7] Srivastava S., Bhargava A. (2016). Biofilms and human health. Biotechnol. Lett..

[8] Kamaruzzaman N.F., Firdessa R., Good L. (2016). Bactericidal effects of polyhexamethylene biguanide against intracellular *Staphylococcus aureus* EMRSA-15 and USA 300. J. Antimicrob. Chemoth..

[9] Rasigade J.P., Moulay A., Lhoste Y., Tristan A., Bes M., Vandenesch F., Etienne J., Lina G., Laurent F., Dumitrescu O. (2011). Impact of sub-inhibitory antibiotics on fibronectin-mediated host cell adhesion and invasion by *Staphylococcus aureus*. BMC Microbiol..

[10] Loomba P.S., Taneja J., Mishra B. (2010). Methicillin and Vancomycin Resistant *S. aureus* in Hospitalized Patients. J. Glob. Infect. Dis..

[11] Rossi F., Diaz L., Wollam A., Panesso D., Zhou Y., Rincon S., Narechania A., Xing G., Di Gioia T.S.R., Doi A. (2014). Transferable Vancomycin Resistance in a Community-Associated MRSA Lineage. N. Engl. J. Med..

[12] Rodvold K.A., McConeghy K.W. (2014). Methicillin-Resistant *Staphylococcus aureus* Therapy: Past, Present, and Future. Clin. Infect. Dis..

[13] Chhibber T., Gondil V.S., Sinha V.R. (2020). Development of Chitosan-Based Hydrogel Containing Antibiofilm Agents for the Treatment of *Staphylococcus aureus*-Infected Burn Wound in Mice. AAPS Pharmscitech.

[14] Wong-Beringer A., Joo J., Tse E., Beringer P. (2011). Vancomycin-associated nephrotoxicity: A critical appraisal of risk with high-dose therapy. Int. J. Antimicrob. Agents.

[15] Waterer G., Lord J., Hofmann T., Jouhikainen T. (2020). Phase I, Dose-Escalating Study of the Safety and Pharmacokinetics of Inhaled Dry-Powder Vancomycin (AeroVanc) in Volunteers and Patients with Cystic Fibrosis: A New Approach to Therapy for Methicillin-Resistant *Staphylococcus aureus*. Antimicrob. Agents Chemother..

[16] Worthington R.J., Melander C. (2013). Combination approaches to combat multidrug-resistant bacteria. Trends Biotechnol..

[17] Dolgin E. (2010). Sequencing of superbugs seen as key to combating their spread. Nat. Med..

[18] Walsh C. (2000). Molecular mechanisms that confer antibacterial drug resistance. Nature.

[19] Tamma P.D., Cosgrove S.E., Maragakis L.L. (2012). Combination therapy for treatment of infections with gram-negative bacteria. Clin. Microbiol. Rev..

[20] Thompson J.M., Saini V., Ashbaugh A.G., Miller R.J., Ordonez A.A., Ortines R.V., Wang Y., Sterling R.S., Jain S.K., Miller L.S. (2017). Oral-Only Linezolid-Rifampin Is Highly Effective Compared with Other Antibiotics for Periprosthetic Joint Infection Study of a Mouse Model. J. Bone Joint Surg. Am..

[21] Petrosillo N., Ioannidou E., Falagas M.E. (2008). Colistin monotherapy vs. combination therapy: Evidence from microbiological, animal and clinical studies. Clin. Microbiol. Infect..

[22] Crunkhorn S. (2016). Combination therapy combats MRSA. Nat. Rev. Drug Discov..

[23] Yang H., Zhang H., Wang J., Yu J., Wei H. (2017). A novel chimeric lysin with robust antibacterial activity against planktonic and biofilm methicillin-resistant *Staphylococcus aureus*. Sci. Rep..

[24] Miro-Canturri A., Ayerbe-Algaba R., Jimenez-Mejias M.E., Pachon J., Smani Y. (2021). Efficacy of Lysophosphatidylcholine as Direct Treatment in Combination with Colistin against *Acinetobacter baumannii* in Murine Severe Infections Models. Antibiotics.

[25] Xu N., Cheng H., Xu J.W., Li F., Gao B.A., Li Z., Gao C.H., Huo K.F., Fu J.J., Xiong W. (2017). Silver-loaded nanotubular structures enhanced bactericidal efficiency of antibiotics with synergistic effect in vitro and in vivo. Int. J. Nanomedicine.

[26] Cheng K.Y., Lin Z.H., Cheng Y.P., Chiu H.Y., Yeh N.L., Wu T.K., Wu J.S. (2018). Wound Healing in Streptozotocin-Induced Diabetic Rats Using Atmospheric-Pressure Argon Plasma Jet. Sci. Rep..

[27] Xu H., Ma R., Zhu Y., Du M., Zhang H., Jiao Z. (2020). A systematic study of the antimicrobial mechanisms of cold atmospheric-pressure plasma for water disinfection. Sci. Total Environ..

[28] Ma M.Y., Zhang Y.Z., Lv Y., Sun F.S. (2020). The key reactive species in the bactericidal process of plasma activated water. J. Phys. D Appl. Phys..

[29] Thirumdas R., Kothakota A., Annapure U., Siliveru K., Blundell R., Gatt R., Valdramidis V.P. (2018). Plasma activated water (PAW): Chemistry, physico-chemical properties, applications in food and agriculture. Trends Food Sci. Tech..

[30] Li D., Liu D., Nie Q., Li H., Chen H., Kong M.G. (2014). Array of surface-confined glow discharge at atmospheric helium: Modes and dynamics. Appl. Phys. Lett..

[31] Zhou R., Zhou R., Prasad K., Fang Z., Speight R., Bazaka K., Ostrikov K. (2018). Cold atmospheric plasma activated water as a prospective disinfectant: The crucial role of peroxynitrite. Green Chem..

[32] Zhou R., Zhou R., Wang P., Xian Y., Mai-Prochnow A., Lu X., Cullen P.J., Ostrikov K., Bazaka K. (2020). Plasma-activated water: Generation, origin of reactive species and biological applications. J. Phys. D Appl. Phys..

[33] Kong M.G., Deng X.T. (2003). Electrically efficient production of a diffuse nonthermal atmospheric plasma. IEEE Trans. Plasma Sci..

[34] Liu Z., Liu D., Chen C., Li D., Yang A., Rong M., Chen H.L., Kong M.G. (2015). Physicochemical processes in the indirect interaction between surface air plasma and deionized water. J. Phys. D Appl. Phys..

[35] Gorbanev Y., O’Connell D., Chechik V. (2016). Ozone correlates with antibacterial effects from indirect air dielectric barrier discharge treatment of water. Chem. Eur. J..

[36] Shen J., Tian Y., Li Y.L., Ma R.A., Zhang Q., Zhang J., Fang J. (2016). Bactericidal Effects against *S-aureus* and Physicochemical Properties of Plasma Activated Water stored at different temperatures. Sci. Rep..

[37] Balan G.G., Rosca I., Ursu E.L., Doroftei F., Bostanaru A.C., Hnatiuc E., Nastasa V., Sandru V., Stefanescu G., Trifan A. (2018). Plasma-activated water: A new and effective alternative for duodenoscope reprocessing. Infect. Drug Resist..

[38] Laurita R., Contaldo N., Zambon Y., Bisag A., Canel A., Gherardi M., Laghi G., Bertaccini A., Colombo V. (2021). The use of plasma-activated water in viticulture: Induction of resistance and agronomic performance in greenhouse and open field. Plasma Process. Polym..

[39] Wandell R.J., Locke B.R. (2014). Hydrogen Peroxide Generation in Low Power Pulsed Water Spray Plasma Reactors. Ind. Eng. Chem. Res..

[40] Zhang Q., Ma R.N., Tian Y., Su B., Wang K.L., Yu S., Zhang J., Fang J. (2016). Sterilization Efficiency of a Novel Electrochemical Disinfectant against *Staphylococcus aureus*. Environ. Sci. Technol..

[41] Vlad I.E., Martin C., Toth A.R., Papp J., Anghel S.D. (2019). Bacterial Inhibition Effect of Plasma Activated Water. Rom. Rep. Phys..

[42] Charoux C.M.G., Patange A.D., Hinds L.M., Simpson J.C., O’Donnell C.P., Tiwari B.K. (2020). Antimicrobial effects of airborne acoustic ultrasound and plasma activated water from cold and thermal plasma systems on biofilms. Sci. Rep..

[43] Su X., Tian Y., Zhou H.Z., Li Y.L., Zhang Z.H., Jiang B.Y., Yang B., Zhang J., Fang J. (2018). Inactivation Efficacy of Nonthermal Plasma-Activated Solutions against *Newcastle* Disease Virus. Appl. Environ. Microbiol..

[44] Zhang J.Y., Qu K., Zhang X., Wang B.C., Wang W.T., Bi J.B., Zhang S.M., Li Z.Y., Kong M.G., Liu D.X. (2019). Discharge Plasma-Activated Saline Protects against Abdominal Sepsis by Promoting Bacterial Clearance. Shock.

[45] Takeda S., Yamada S., Hattori N., Nakamura K., Tanaka H., Kajiyama H., Kanda M., Kobayashi D., Tanaka C., Fujii T. (2017). Intraperitoneal Administration of Plasma-Activated Medium: Proposal of a Novel Treatment Option for Peritoneal Metastasis From Gastric Cancer. Ann. Surg. Oncol..

[46] Guo L., Xu R.B., Zhao Y.M., Liu D.X., Liu Z.J., Wang X.H., Chen H.L., Kong M.G. (2018). Gas Plasma Pre-treatment Increases Antibiotic Sensitivity and Persister Eradication in Methicillin-Resistant *Staphylococcus aureus*. Front. Microbiol..

[47] Cheng J., Chin W., Dong H., Xu L., Zhong G., Huang Y., Li L., Xu K., Wu M., Hedrick J.L. (2015). Biodegradable Antimicrobial Polycarbonates with In Vivo Efficacy against Multidrug-Resistant MRSA Systemic Infection. Adv. Healthc. Mater..

[48] Otto M. (2012). Methicillin-resistant *Staphylococcus aureus* infection is associated with increased mortality. Future Microbiol..

[49] Ho P.L., Lo P.Y., Chow K.H., Lau E.H., Lai E.L., Cheng V.C., Kao R.Y. (2010). Vancomycin MIC creep in MRSA isolates from 1997 to 2008 in a healthcare region in Hong Kong. J. Infect..

[50] Tsiodras S., Gold H.S., Sakoulas G., Eliopoulos G.M., Wennersten C., Venkataraman L., Moellering R.C., Ferraro M.J. (2001). Linezolid resistance in a clinical isolate of *Staphylococcus aureus*. Lancet.

[51] Van Hal S.J., Lodise T.P., Paterson D.L. (2012). The Clinical Significance of Vancomycin Minimum Inhibitory Concentration in *Staphylococcus aureus* Infections: A Systematic Review and Meta-analysis. Clin. Infect. Dis..

[52] Melo-Cristino J., Resina C., Manuel V., Lito L., Ramirez M. (2013). First case of infection with vancomycin-resistant *Staphylococcus aureus* in Europe. Lancet.

[53] Hua X., Yang Q., Zhang W., Dong Z., Yu S., Schwarz S., Liu S. (2018). Antibacterial Activity and Mechanism of Action of Aspidinol Against Multi-Drug-Resistant Methicillin-Resistant *Staphylococcus aureus*. Front. Pharmacol..

[54] Tacconelli E., Carrara E., Savoldi A., Harbarth S., Mendelson M., Monnet D.L., Pulcini C., Kahlmeter G., Kluytmans J., Carmeli Y. (2018). Discovery, research, and development of new antibiotics: The WHO priority list of antibiotic-resistant bacteria and tuberculosis. Lancet Infect. Dis..

[55] Sobieraj A.M., Huemer M., Zinsli L.V., Meile S., Keller A.P., Rohrig C., Eichenseher F., Shen Y., Zinkernagel A.S., Loessner M.J. (2020). Engineering of Long-Circulating Peptidoglycan Hydrolases Enables Efficient Treatment of Systemic *Staphylococcus aureus* Infection. Mbio.

[56] Ajiboye T.O. (2019). Contributions of reactive oxygen species, oxidative DNA damage and glutathione depletion to the sensitivity of *Acinetobacter baumannii* to 2-(2-nitrovinyl) furan. Microb. Pathog..

[57] Tasaki M., Kuroiwa Y., Inoue T., Hibi D., Matsushita K., Ishii Y., Maruyama S., Nohmi T., Nishikawa A., Umemura T. (2013). Oxidative DNA damage and in vivo mutagenicity caused by reactive oxygen species generated in the livers of p53-proficient or -deficient gpt delta mice treated with non-genotoxic hepatocarcinogens. J. Appl.Toxicol..

[58] He L.-L., Wang X., Wu X.-X., Wang Y.-X., Kong Y.-M., Wang X., Liu B.-M., Liu B. (2015). Protein damage and reactive oxygen species generation induced by the synergistic effects of ultrasound and methylene blue. Spectrochim. Acta Part A Mol. Biomol. Spectrosc..

[59] Downs D.M., Ernst D.C. (2015). From microbiology to cancer biology: The Rid protein family prevents cellular damage caused by endogenously generated reactive nitrogen species. Mol. Microbiol..

[60] Xu D.H., Wang S., Li B., Qi M., Feng R., Li Q.S., Zhang H., Chen H.L., Kong M.G. (2020). Effects of Plasma-Activated Water on Skin Wound Healing in Mice. Microorganisms.

[61] Zou X.Y., Xu M.Y., Pan S.H., Gan L., Zhang S., Chen H.X., Liu D.W., Lu X.P., Ostrikov K.K. (2019). Plasma Activated Oil: Fast Production, Reactivity, Stability, and Wound Healing Application. ACS Biomater. Sci. Eng..

[62] Yousfi M., Merbahi N., Pathak A., Eichwald O. (2014). Low-temperature plasmas at atmospheric pressure: Toward new pharmaceutical treatments in medicine. Fundam. Clin. Pharmacol..

[63] Azzariti A., Iacobazzi R.M., Di Fonte R., Porcelli L., Gristina R., Favia P., Fracassi F., Trizio I., Silvestris N., Guida G. (2019). Plasma-activated medium triggers cell death and the presentation of immune activating danger signals in melanoma and pancreatic cancer cells. Sci. Rep..

[64] He L., He T., Farrar S., Ji L., Liu T., Ma X. (2017). Antioxidants Maintain Cellular Redox Homeostasis by Elimination of Reactive Oxygen Species. Cell. Physiol. Biochem..

[65] Zhang R., Zhao J., Han G., Liu Z., Liu C., Zhang C., Liu B., Jiang C., Liu R., Zhao T. (2016). Real-Time Discrimination and Versatile Profiling of Spontaneous Reactive Oxygen Species in Living Organisms with a Single Fluorescent Probe. J. Am. Chem. Soc..

[66] Xie Y., Xianyu Y., Wang N., Yan Z., Liu Y., Zhu K., Hatzakis N.S., Jiang X. (2018). Functionalized Gold Nanoclusters Identify Highly Reactive Oxygen Species in Living Organisms. Adv. Funct. Mater..

[67] Guo L., Xu R.B., Liu D.X., Qi Y., Guo Y.H., Wang W.T., Zhang J., Liu Z.J., Kong M.G. (2019). Eradication of methicillin-resistant *Staphylococcus aureus* biofilms by surface discharge plasmas with various working gases. J. Phys. D Appl. Phys..

