# Supplementary material for: Plasma-Activated Saline Promotes Antibiotic Treatment of Systemic Methicillin-Resistant Staphylococcus aureus Infection
Source: Antibiotics (Basel). 2021 Aug 22;10(8):1018. doi: 10.3390/antibiotics10081018 (PMC8388904; doi:10.3390/antibiotics10081018)

antibiotics

MDPI

Article

Plasma-Activated Saline Promotes Antibiotic Treatment of Systemic Methicillin-Resistant *Staphylococcus aureus* Infection

Lu Yang [1], Gulimire Niyazi [1], Yu Qi [2], Zhiqian Yao [2], Lingling Huang [2], Zifeng Wang [2], Li Guo [2,*] and Dingxin Liu [2,*]

[1] School of Life Science and Technology, Xi'an Jiaotong University, Xi'an 710049, China; yanglu35@stu.xjtu.edu.cn (L.Y.); gulmira@stu.xjtu.edu.cn (G.N.)

[2] State Key Laboratory of Electrical Insulation and Power Equipment, Center for Plasma Biomedicine, Xi'an Jiaotong University, Xi'an 710049, China; qq460820339@stu.xjtu.edu.cn (Y.Q.); yaozhiqian0512@stu.xjtu.edu.cn (Z.Y.); huangll@stu.xjtu.edu.cn (L.H.); zsdgsd@stu.xjtu.edu.cn (Z.W.)

* Correspondence: guoli35@mail.xjtu.edu.cn (L.G.); liudingxin@mail.xjtu.edu.cn (D.L.)

Figure S1. Inactivation of MRSA biofilms by the combination of the reconstituted saline solution (30 μM H_2O_2, 2.5 μM NO_2^-, and 6500 μM NO_3^- in saline solution) and antibiotics in vitro. The reconstituted saline solution-treated and untreated biofilms were incubated with TSB broth containing vancomycin or rifampicin. The treated and untreated biofilms were solubilized in 1 mL PBS by sonication and vortexing. Serial dilutions of each biofilm were performed and 10 μL of each dilution was spotted onto TSB plates and incubated overnight at 37°C. The resulting colonies were counted and analyzed. Error bars represent the standard deviation (SD).

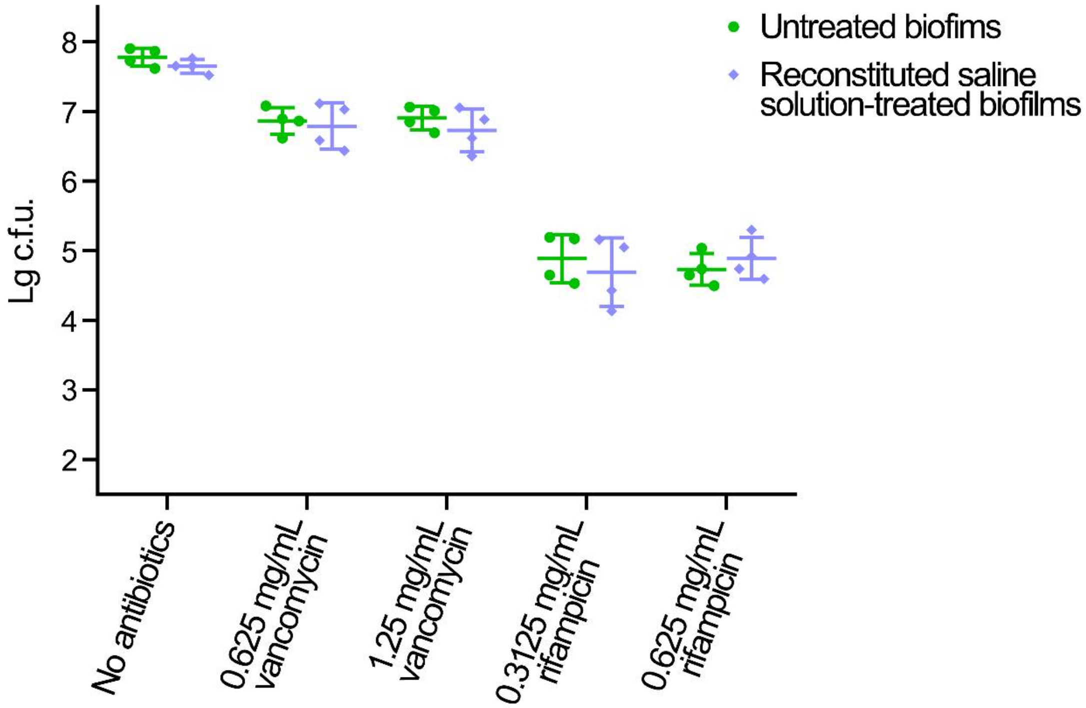

Figure S2. Evaluation of the numbers of MRSA cells in different organs from the murine systemic model. Groups of 5 Balb/c mice were used. Colony-forming units (c.f.u.) from the heart, liver, spleen, lung, and kidney of each mouse were plotted as individual points and error bars represent the standard deviation (SD) within an experimental group. Black circles, hearts; green squares, livers; orange regular triangles, spleen; red inverted triangles, lungs; blue rhombuses, left kidneys; purple circles, right kidneys.

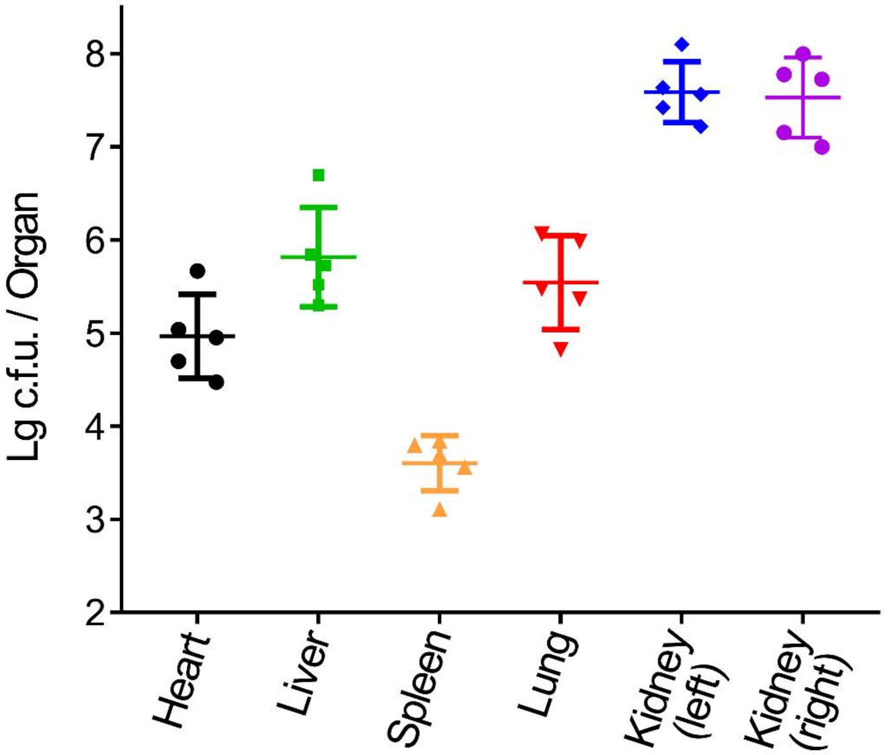

Figure S3. Treatment of MRSA systemic infection by the combination of the reconstituted saline solution (30 μM H_2O_2, 2.5 μM NO_2^-, and 6500 μM NO_3^- in saline solution) and rifampicin in the murine model. Groups of 5 Balb/c mice were used. Colony-forming units (c.f.u.) from one kidney of each mouse were plotted as individual points and error bars represent the standard deviation (SD) within an experimental group. Black squares, untreated group; lavender regular triangles, the group treated with the reconstituted saline solution; red inverted triangles, the group treated with rifampicin; khaki rhombuses, the group treated with the combination of the reconstituted saline solution and rifampicin.

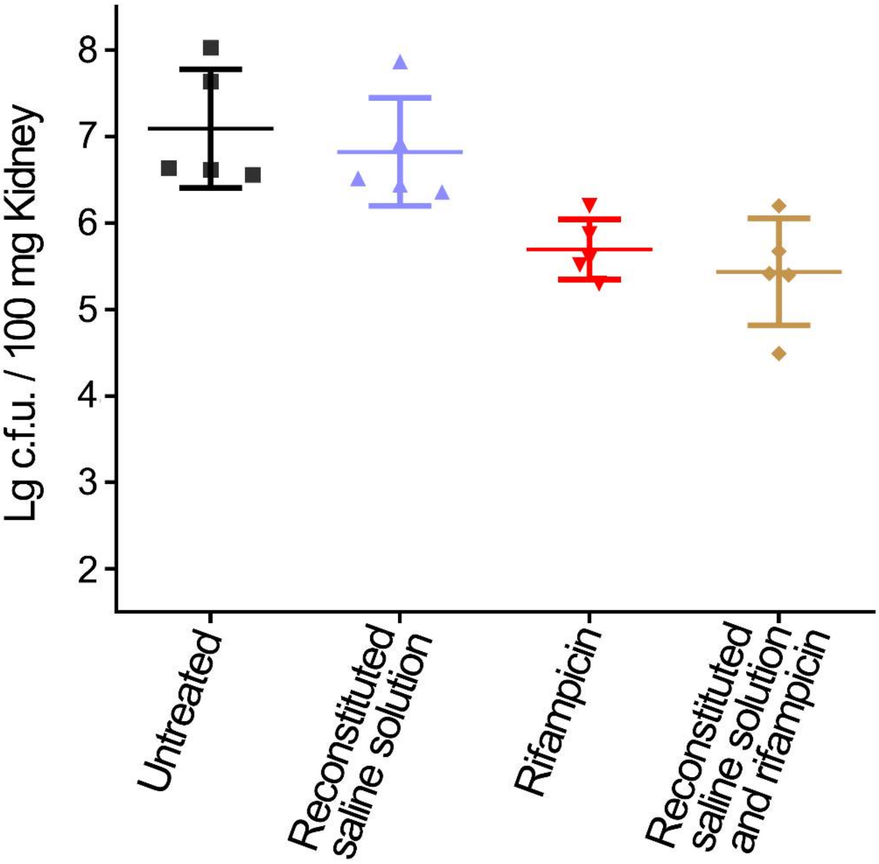

Supplement: Supplementary file 1 [file antibiotics-10-01018-s001.zip › antibiotics-1316202-supplementary.pdf]
